# The Complex Relationship between Antipsychotic-Induced Weight Gain and Therapeutic Benefits: A Systematic Review and Implications for Treatment

**DOI:** 10.3389/fnins.2017.00741

**Published:** 2018-01-22

**Authors:** Alex T. Raben, Victoria S. Marshe, Araba Chintoh, Ilona Gorbovskaya, Daniel J. Müller, Margaret K. Hahn

**Affiliations:** ^1^Schizophrenia Program, Centre for Addiction and Mental Health, Toronto, ON, Canada; ^2^Department of Psychiatry, Faculty of Medicine, University of Toronto, Toronto, ON, Canada; ^3^Pharmacogenetic Research Clinic, Campbell Family Mental Health Research Institute, Centre for Addiction and Mental Health, Toronto, ON, Canada; ^4^Institute of Medical Science, Faculty of Medicine, University of Toronto, Toronto, ON, Canada

**Keywords:** antipsychotics, metabolic dysregulation, antipsychotic-induced weight gain (AIWG), treatment outcome, weight loss, 82453 (PROSPERO).

## Abstract

**Background:** Antipsychotic-induced weight gain (AIWG) and other adverse metabolic effects represent serious side effects faced by many patients with psychosis that can lead to numerous comorbidities and which reduce the lifespan. While the pathophysiology of AIWG remains poorly understood, numerous studies have reported a positive association between AIWG and the therapeutic benefit of antipsychotic medications.

**Objectives:** To review the literature to (1) determine if AIWG is consistently associated with therapeutic benefit and (2) investigate which variables may mediate such an association.

**Data Sources:** MEDLINE, Google Scholar, Cochrane Database and PsycINFO databases were searched for articles containing all the following exploded MESH terms: schizophrenia [AND] antipsychotic agents/neuroleptics [AND] (weight gain [OR] lipids [OR] insulin [OR] leptin) [AND] treatment outcome. Results were limited to full-text, English journal articles.

**Results:** Our literature search uncovered 31 independent studies which investigated an AIWG-therapeutic benefit association with a total of 6063 enrolled individuals diagnosed with schizophrenia or another serious mental illness receiving antipsychotic medications. Twenty-two studies found a positive association while, 10 studies found no association and one study reported a negative association. Study variables including medication compliance, sex, ethnicity, or prior antipsychotic exposure did not appear to consistently affect the AIWG-therapeutic benefit relationship. In contrast, there was some evidence that controlling for baseline BMI/psychopathology, duration of treatment and specific agent studied [i.e., olanzapine (OLZ) or clozapine (CLZ)] strengthened the relationship between AIWG and therapeutic benefit.

**Limitations:** There were limitations of the reviewed studies in that many had small sample sizes, and/or were retrospective. The heterogeneity of the studies also made comparisons difficult and publication bias was not controlled for.

**Conclusions:** An AIWG-therapeutic benefit association may exist and is most likely to be observed in OLZ and CLZ-treated patients. The clinical meaningfulness of this association remains unclear and weight gain and other metabolic comorbidities should be identified and treated to the same targets as the general population. Further research should continue to explore the links between therapeutic benefit and metabolic health with emphasis on both pre-clinical work and well-designed prospective clinical trials examining metabolic parameters associated, but also occurring independently to AIWG.

## Introduction

Second generation antipsychotics (SGAs; also known as atypical antipsychotics) are the cornerstone of schizophrenia treatment. They effectively reduce psychotic symptoms while demonstrating a reduction in extrapyramidal side effects as compared to their typical, or first generation counterparts. However, SGAs are not without their own set of common side effects. Numerous studies have linked SGAs to metabolic changes which place patients at risk for cardiovascular complications, including antipsychotic-induced weight gain (AIWG), dyslipidemia, insulin resistance and type-2 diabetes (Wirshing et al., 1998; Allison and Casey, 2001; Nasrallah, 2003; Nasrallah and Newcomer, 2004; Mackin et al., 2005; Chiu et al., 2006; Oriot et al., 2008; Fernandez-Egea et al., 2011). Notably, patients with schizophrenia have significantly reduced lifespans compared to the general population—by 11–20 years—which is attributable to their higher risk of death from cardiovascular causes (Hennekens and Newcomer, 2007; Laursen et al., 2013; Kredentser et al., 2014).

The metabolic liability differs among agents, with clozapine (CLZ), and olanzapine (OLZ), representing the two SGAs with the most adverse metabolic profile. Despite differences among agents, even the SGAs considered to be most metabolically neutral, (e.g., ziprasidone, and aripiprazole), are associated with significant weight gain in antipsychotic antipsychotic-naïve individuals (Correll, 2009; Patel et al., 2009; Bak et al., 2014). Moreover, CLZ represents the sole antipsychotic with superiority in treatment refractory schizophrenia, with some evidence also supporting efficacy advantages for OLZ, precluding avoidance of these agents in severely ill individuals (Lieberman et al., 2005). Therefore, with existing antipsychotics, early and effective treatment of schizophrenia is associated with an increase in cardiovascular burden.

Despite the associated cardio-metabolic morbidity, the underlying causes of AIWG and other metabolic changes remain largely elusive. Increased food intake appears to be a component (Benarroch et al., 2016), although it is less clear if AIWG is also attributable to reductions in resting energy expenditure (Cuerda et al., 2013). Animal and some human studies show that the various areas of neurotransmission altered by antipsychotics may affect energy and glucose regulation (Hahn et al., 2011). Antagonism of the histamine H1 (H1), serotonin 2A/2C (5-HT2A/C), and dopamine D1/D2/D3 receptors, as well as, adrenergic and muscarinic receptors are common effects of antipsychotics and each receptor has been implicated in weight regulation (Roerig et al., 2011). Similarly, disruption of insulin homeostasis has been tied to antagonism of serotonergic (5-HT2A/C) (Nonogaki et al., 1998; Gilles et al., 2005; Tulipano et al., 2007; Hahn et al., 2011; Guenette et al., 2013), adrenergic (α1) (Zillich et al., 2006; Savoy et al., 2008; Guenette et al., 2013) and muscarinic (M3) receptors (Hahn et al., 2011), both via the peripheral and central nervous system (CNS). Taken together, the possibility exists that neurotransmitter systems implicated in energy homeostasis may overlap with those implicated in therapeutic action of antipsychotics.

Notably, a large number of studies have studied the genetics of AIWG in order to identify subjects at high risk, unravel mechanisms and suggest new therapeutic drug targets. For AIWG and other antipsychotic-induced metabolic dysregulation, genetic variation of the dopamine D2 receptor (*DRD2*) and 5-HT2c receptor (*HTR2C*) genes have been substantially investigated. Although *DRD2* variants have been associated with improvement of psychopathology (Zhang et al., 2010, 2015; Huang et al., 2016), those variants have not been consistently found to be associated with AIWG (Lett et al., 2011; Shams and Müller, 2014). For *HTR2C*, although variants have been implicated in AIWG, there is limited evidence linking these variants to therapeutic benefit (Lett et al., 2011; Mueller, 2011). At the presynaptic level, the synaptosomal-associated protein of 25 kDa (SNantipsychotic-25) which mediates presynaptic vesicle trafficking has been reported to be associated with AIWG and therapeutic benefit in a smaller study (Müller et al., 2005). This finding is of particular interest to insulin secretion which is mediated through SNantipsychotic-25 in the pancreas.

With respect to insulin-related pathways, genes including leptin, neuropeptide-Y (*NPY*) and glucagon-like peptide-1 (*GLP-1*) were reported to be associated with AIWG. Leptin plays a role in regulating circulating insulin (Yadav et al., 2013) and may moderate severity of clinical symptoms (Takayanagi et al., 2013; Nurjono et al., 2014). *LEP* rs7799039 has been associated with AIWG in 451 patients (Shen et al., 2014). NPY is a pancreatic neuropeptide expressed in the arcuate nucleus of the hypothalamus and stimulates food intake (Sato, 2005). *NPY* rs16147 has been significantly associated with AIWG in OLZ- and CLZ- treated patients; Tiwari et al., 2013), while other variants have been associated with symptoms and cortical structure in schizophrenia patients (LaCrosse and Olive, 2013). Lastly, GLP-1 (encoded by the *GCG* gene) is a gastric hormone which stimulates the secretion of insulin. Previous associations have been reported between AIWG and *GCG* rs13429709 in OLZ- or CLZ- treated patients, as well as, the *SULT4A1-1* haplotype in OLZ-treated patients from the CATIE trial for which patients had greater clinical response but gained significantly less weight (Ramsey and Brennan, 2014).

Other genes which have been investigated in association with AIWG (particularly by studying CLZ and OLZ) include *cannabinoid receptor 1* (*CNR1*) (Tiwari et al., 2010), *ghrelin* (*GHRL*) (Yang et al., 2012), *leptin receptor* (*LEPR*) (Gregoor et al., 2009; Perez-Iglesias et al., 2010; Brandl et al., 2012), *fat-mass and obesity-associated protein* (*FTO*) (Jassim et al., 2010; Perez-Iglesias et al., 2010; Reynolds et al., 2012; Shing et al., 2014), *adiponectin* (*ADIPOQ*) (Jassim et al., 2010; Brandl et al., 2014), as well as, *insulin-induced genes 1 and 2* (INSIG1 and INSIG2) (Le Hellard et al., 2008).

However, the most consistent genetic evidence has been presented for the melanocortin-4 receptor (*MC4R*). *MC4R* has been implicated in appetitive behavior, as well as, metabolic and weight regulation (Santini et al., 2009; Chowdhury et al., 2012; Malhotra, 2012; Yilmaz et al., 2014). *MC4R* remains the leading gene associated with monogenic obesity (Loos et al., 2008) and insulin resistance (Chambers et al., 2008). In particular for AIWG, a marker near the *MC4R gene locus* (rs489693) has been associated with SGA-related AIWG in at least three independent samples (Shams and Müller, 2014).

To summarize the large number of genetic studies conducted in AIWG, it appears that genes in distinct pathways or systems (i.e., at the receptor, synapse and energy homeostasis level) might be associated with AIWG, with each of them contributing limited risk, as expected in complex phenotypes. While there might be genetic variants which contribute to AIWG and therapeutic benefit simultaneously, there is a paucity of studies addressing this relationship and questions remain if AIWG and therapeutic benefit operate independently, i.e., where AWIG would be a pure side effect without therapeutic benefit.

In this review, we set out to evaluate whether an association between AIWG and/or other antipsychotic-related metabolic effects (e.g., insulin resistance, dyslipidemia) exists in relation to the therapeutic benefit of antipsychotics, across illness stage (also a proxy of age), ethnicities and study designs. We also discuss evidence whether such an association could be spurious vs. a true phenomenon with clinical implications. If indeed a true association exists between antipsychotic-induced metabolic dysregulation and treatment response, this would carry important clinical implications, such as the ability to safely target metabolic side-effects without compromise of treatment response, and the development of metabolically neutral yet effective drugs.

## Methods

To answer our research questions, we conducted a systematic review of studies investigating the association between therapeutic benefit and AIWG. We searched for relevant systematic review databases, including PROSPERO and the Cochrane Database, and found no similar reviews on this topic. We also followed the PRISMA guidelines and completed a PRISMA checklist (see Supplementary Table 1).

We searched MEDLINE, Google Scholar and PsycINFO databases for articles containing all the following exploded MESH terms combine in the following way: *schizophrenia* [AND] *antipsychotic agents/neuroleptics* [AND] (*weight gain* [OR] *lipids* [OR] *insulin* [OR] *leptin*) [AND] *treatment outcome*. Results were limited to full-text, English journal articles that studied humans up to a publication date of November 2016. The resulting abstracts were reviewed by ATR and MKH and those articles of relevance were assembled. Each of these articles was then hand-searched by ATR and MKH and only those that investigated the association between AIWG or antipsychotic-induced metabolic changes (e.g., change in insulin levels) and therapeutic benefit either by correlation or comparison were included in further analysis. The bibliographies of the remaining articles were then hand-searched by ATR for further relevant articles and any of these additional articles which investigated for an AIWG/metabolic changes and therapeutic benefit link were included in our analysis. Finally, articles that did not perform a statistical analysis were excluded from our analysis.

Our primary outcome of interest was the examination of a correlation between AIWG metabolic changes and therapeutic benefit. A study population was considered to provide evidence for an AIWG/metabolic changes and therapeutic benefit association when patients treated with an antipsychotic showed a statistically significant (*p* < 0.05) correlation between AIWG and therapeutic benefit or a statistically significant (*p* < 0.05) difference in measures of therapeutic benefit relative to weight gain/metabolic changes. In addition, we performed an exploratory analysis to investigate clinical and demographic factors which might impact the association between AIWG and therapeutic benefit to identify potential mechanisms and mitigating factors.

## Results

**Figure 1:**
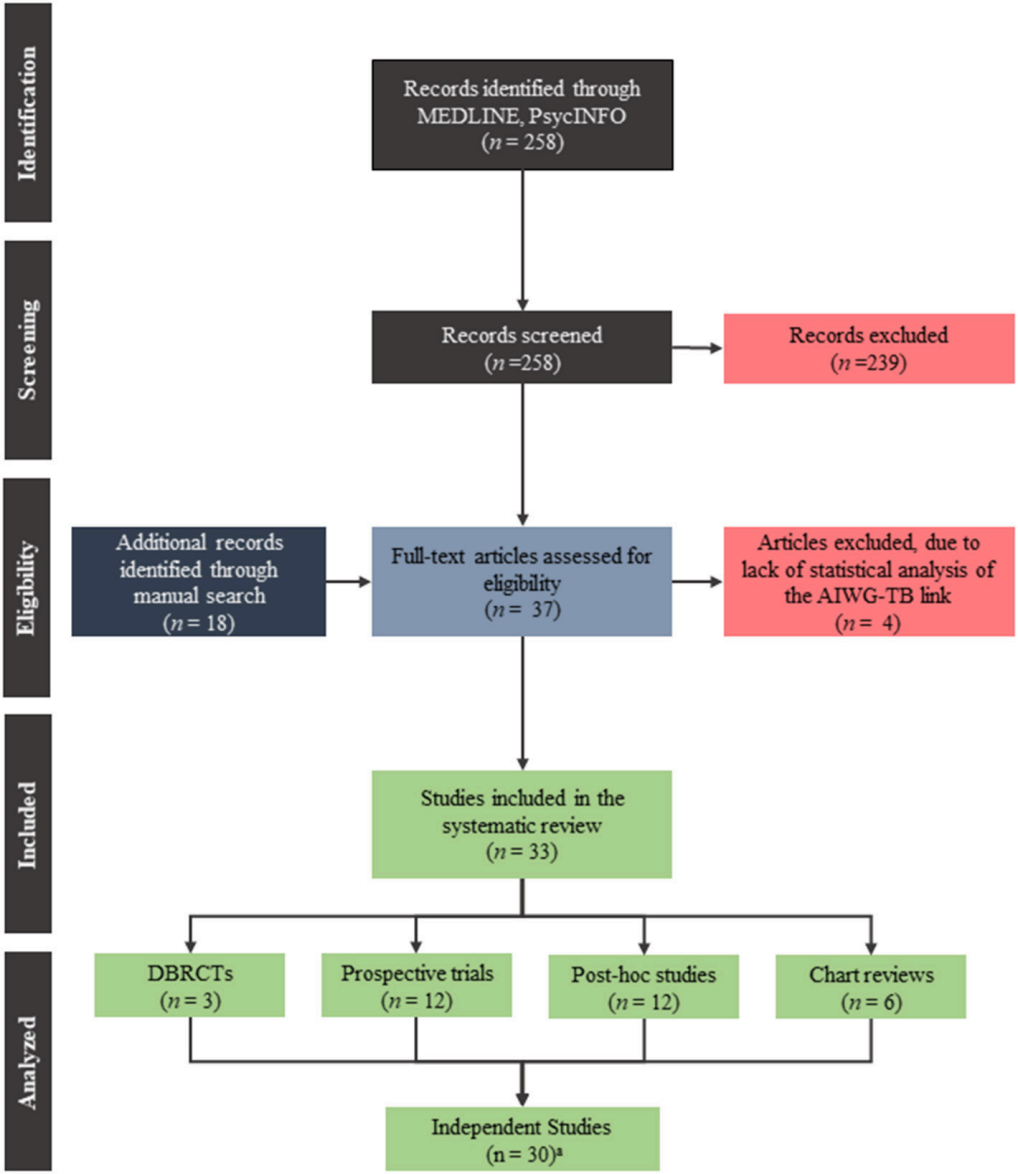
PRISMA flow diagram of the search used to find the publications included in this systematic review.

Our search retrieved 37 articles which investigated the link between metabolic changes and psychopathology in schizophrenia patients treated with antipsychotics. Of those, four studies were excluded (Gordon and Groth, 1964; Singh et al., 1970; Gupta et al., 1999; Wetterling and Müssigbrodt, 1999) due to lack of statistical analyses examining the AIWG-therapeutic benefit link. See Figure 1 for a flow diagram of the search and Table 1 for a summary of the 33 articles included in this review.

**Table 1:** Summary of the 33 articles included in this review.

**Reference**	**Population**	***N***	**Treatment**	**Duration (weeks)**	**Measures**	**Results**
**Double-Blind Randomized Controlled Trials (DBRCTs)**						
Planansky and Heilizer, 1959^b^	SCZ (male)^a^	59	CPZ (*N* = 33)	12	Jump reaction time;	- CPZ: Jump Time Consistency (*r* = 0.46; *p* = 0.05) and A cluster, increased activity (*r* = 0.70; *p* = 0.01).
			PBO (*N* = 26)		A/F clusters (Lorr Scale)	
						- PBO: No significant associations.
Holden and Holden, 1970^b^	SCZ (male)^a^	22	CDX	8	7-point global scale	- % WG significantly correlated with decreased associative and perceptual disturbance, motor activity, depression, emotional liability and confusion (*r* = 0.43; *p* < 0.01, *R^2^* = 0.12)
			TDZ		BPRS	
			PBO		MIPRS	
						- CDX: % WG significantly correlated with increased drive
						- TDZ: % WG significantly correlated with decreased associative disturbance, agitation, drive and confusion
						- CDX + TDZ: % WG significantly correlated with decreased confusion, thinking disturbance and paranoid reaction
						- PBO: % WG significantly correlated with decreased associative and perceptual disturbance, motor activity, apathy, confusion, thinking disturbance and paranoid reaction (*r* = 0.47; *p* < 0.01, *R^2^* = 0.16)
**Poyurovsky et al.**, **2002**	SCZ^a^	30	OLZ+PBO (*N* = 15)	8	SANS	- OLZ+PBO: No significant association with pos. (*r* = 0.45), neg. (*r* = 0.17) or disorganize (*r* = 0.20) symptoms
			OLZ+FLX (*N =* 15)		SAPS	
						- OLZ+FLX: No significant association with pos. (*r* = 0.08), neg. (*r* = 0.21) or disorganize (*r* = 0.11) symptoms
**PROSPECTIVE STUDIES**						
Leadbetter et al., 1992^b^	SCZ	21	CLZ	16	BPRS	- Significant association of BRPS score with WG (*r* = 0.54, *p* = 0.01)
	SAD					
						- TB difference between marked WG (≥10% increase) and non-marked WG (10.2 BPRS total points, *p* < 0.03; neg. symptoms, 3.5 points, *p* < 0.02).
Jalenques et al., 1996	SCZ	15	CLZ	84	BPRS	- All patients showed same improvement at 20 weeks.
						- Group 1: Achieved further improvement (maximally, 8% further decrease in BPRS) and had WG: (12.4 kg; *p* < 0.001
						- Group 2: Worsened (at worst BPRS of 89% initial improvement) and had NS WG on average.
**Hummer et al.**, **1995**	SCZ	81	CLZ	NR	CGI	- No correlation between CS-WG (≥10% increase) and TB (statistics NR).
Bai et al., 1999^b^	SCZ	96	CLZ	56 ± 24.8	CGI	- Females (*N* = 46): Responders vs. non-responders: 10.9 kg vs. 4.3kg; *p* = 0.002.
						- Males (*N* = 50): Responders vs. non-responders: 7.10 kg vs. 7.10 kg; *p* = NS
Meltzer et al., 2003^b^	SCZ	74	CLZ	24	BPRS	- Controlled for age, baseline psychopathology and baseline weight (no effect of sex)
	SAD				SADS-C	
						- At 6 weeks, % WG correlated with total BPRS (β = 0.28, *p* = 0.05), BPRS pos. symptoms (β = 0.17, *p* = 0.01), SADS-C severity of delusions (β = 0.03, *p* = 0.05), SAPS total (β = 0.10, *p* = 0.04)
					GAFS	
					SANS	
					SAPS	
					QLS	- At 24 weeks, % WG correlated with total BPRS (β = 0.20, *p* = 0.01), and SANS (β = 0.13, *p* = 0.002)
Garyfallos et al., 2003^b^	SCZ	50	OLZ (*N* = 25)	8	PANSS	- OLZ: WG correlated with BMI (*r* = 0.69, *p* < 0.01), triglyceride levels (*r* = 0.71, *p* < 0.001) and total cholesterol (*r* = 0.36, *p* > 0.05)
	SAD		RIS (*N* = 25)			
	SPF^a^					
						- RIS: WG not correlated with BMI (*p* > 0.10), triglycerides (*p* > 0.10), or total cholesterol (*p* > 0.05).
Lane et al., 2003^b^	SCZ^a^	146	RIS	6	PANSS	- Controlled for treatment duration, age, sex, type of SCZ, dose and baseline weight
					NOSIE	
						- WG correlated with pos. symptoms (β = 0.06, *p* = 0.009), neg. symptoms (β = 0.08, *p* = 0.0005), cognitive functions (β = 0.08, *p* = 0.003) and NOSIE (β = 0.04, *p* < 0.0002)
						- Significant difference in WG between responders and non-responders (difference = 0.51kg, *p* = 0.007).
**Konarzewska et al.**, **2013**^b^	SCZ (male)	31	OLZ (*N* = 14)	8	PANSS	- OLZ: PANSS score not associated with BMI (*r* = 0.03, *p* = 0.91)
			RIS (*N* = 17)			
						- TB was significantly correlated with increased insulin levels for PANSS-Total, pos., and general symptoms, but not neg. symptoms (*N* = 10).
						- RIS: PANSS score was not associated with BMI (*r* = 0.36, *p* = 0.15);
						- TB was not correlated with insulin levels (*N* = 16)
**Zhang et al.**, **2003**^b^	SCZ	117	CPZ (*N* = 66)	10	PANSS	- No significant association between change in BMI and TB (*p* > 0.05), after controlling for age, sex, illness duration, baseline BMI, baseline psychopathology, dose and *DRD2* genotype
			RIS (*N* = 43)			
			CLZ (*N* = 4)			
			Other (*N* = 4)			
**Zhang et al.**, **2004**^b^	SCZ	46	CPZ	10	PANSS	- No significant correlation of TB with BMI, AIWG, waist-to-hip ratio, or MRI-measures subcutaneous/ intra-abdominal fat
			RIS			
Venkatasubramanian et al., 2010^b^	SCZ	27	OLZ (*N* = 12)	12	SANS	- SANS score significantly correlated with BMI (*r* = 0.40, *p* = 0.04) and leptin levels (*r* = 0.46, *p* = 0.02).
			RIS (*N* = 12)		SAPS	
			FPX (*N =* 3)			
Schwarz et al., 2012^b^	SCZ^a^	77	VAR	6 - 156	PANSS	- Early relapsers (< 33 weeks; *N* = 9) vs. late relapsers (*N* = 9):
						- had lower BMI (4 kg/m^2^, *p* = 0.04), leptin (change ratio = 0.18, *p* = 0.03) proinsulin (change ratio = 0.29; *p* = 0.02) and higher TGF-α (change ratio = 1.85; *p* = 0.018)
						- Lower leptin (Δratio = 0.21; *p* = 0.024) and insulin (Δratio = 0.50; *p* = 0.024) among other molecules but not BMI at time of relapse.
						- Had 1.66 kg/m^2^ lower BMIΔ and less likely to have an increase in leptin, insulin and c-peptide in first 6 weeks.
**CHART REVIEW**						
Planansky, 1958^b^	SCZ	123	CPZ	20	Nurse evaluation	- 13.8% WG with marked improvement, 11.1% WG with minimal improvement and 5.9% WG with no improvement. (*p* < 0.001)
**Lamberti et al.**, **1992**^b^	SCZ	36	CLZ	24	BPRS	- *r* = 0.31; *p* < 0.1 but NS.
**Umbricht et al.**, **1994**	SCZ	84	CLZ	24	BPRS,	- At 6 weeks: *r* = 0.10; *p* = NS, Responders (*N* = 12) did not have significantly more WG than nonresponders (*N* = 44).
					CGI	
						- At 12 weeks (*N* = 26): *r* = 0.28; *p* = NS, Responders (*N* = 10) did not have significantly more WG than nonresponders (*N* = 16).
						- At 24 weeks: Responders (*N* = 19) did not have a higher %WG or a higher cumulative incidence of 5, 10, or 20% WG than nonresponders (*N* = 20) did in first 8 weeks.
Bai, 2006	SCZ (Taiwanese)	140	CLZ	384	CGI	- Controlling for baseline BMI, gender, age, dose, use of other psychotropic drugs: β = 0.297; *p* = 0.007 at 24 weeks (*N* = 85). β = 0.274; *p* = 0.03 at 384 weeks (*N* = 55).
						- Early responders (CGI 1-2 after 14 months) vs. early nonresponders: 13.8 (8.4) kg vs. 4.5 (12) kg; *p* = NR.
						- No sex differences after controlling for baseline BMI.
Hung et al., 2010^b^	SCZ (Taiwanese)	97	RIS	4	MSPI	- Female patients (*N* = 58): β = 3.26; *p* < 0.01. Adjusting for baseline BMI and MSPI: β = 2.51; *p* < 0.01. Male patients (*N* = 39): β = 0.11; *p* > 0.05. Adjusting for baseline BMI and MSPI: β = 0.30; *p* > 0.05.
Sharma et al., 2011	SCZ	46	RIS (41.8%),	30.14 (18.03)	CGI	•[-]*r* = 0.3; *p* = 0.04
			OLZ (27.9%),			
	BAD		CLZ (14%), Others			
***POST-HOC***						
Agid et al., 2013b	SCZ, SAD	94	OLZ	24	GAF	- patients with CS-WG at week 6 had significantly less improvement in GAF scores at week 24 (*p* = 0.031).
			ZIP			
						- Among patients with CS-WG, mean WG was lower in completers compare to those who dropped out prior to 24 weeks (*p* = 0.002).
Bustillo et al., 1996	SCZ	39	CLZ (*N* = 20)	10	BPRS, SANS	- *r* = 0.48; *p* = 0.002 (%BPRS,%WG). No significant correlations with SANS total score or BPRS subscores.
			HAL(*N* = 19)			
						- CLZ: *r* = 0.57; *p* = 0.01 (%BPRS,%WG). No significant correlation found in 48 week open CLZ trial (*N* = 33) follow-up to original study
						- HAL: *r* = 0.19; p = 0.40 (%BPRS,%WG)
Basson et al., 2001 (Study 1)^1^	SCZ, SAD, SPF	1996	OLZ (*N* = 1336),	6	BPRS	- After adjusting for age, dose, gender, baseline BMI, ethnicity, appetite and treatment group: WG difference between responders (BPRS ≤ 18) and nonresponders: ~0.5 kg (p = 0.003).
			HAL (*N* = 660)			
Basson et al., 2001 (Study 2)	SCZ, SAD, SPF	339	OLZ (N = 172), RIS (*N* = 167)	6	BPRS	- After adjusting for age, dose, gender, baseline BMI, ethnicity, appetite and treatment group: WG difference between responders (BPRS ≤ 17) and nonresponders: ~1.5 kg (*p* = 0.001).
Czobor et al., 2002^2b^	SCZ, SAD	151	- OLZ (*N* = 38)	14	PANSS	- OLZ: Controlling for baseline psychopathology and baseline body weight: partial *r* = 0.57; *p* < 0.0003 (total), partial *r* = 0.61; *p* = 0.0001 (general psychopathology), partial *r* = 0.55; *p* = 0.0001 (pos. symptoms), partial r = 0.34; *p* = 0.042 (neg. symptoms). WG difference between responders and deteriorators: 9 kg (*p* = 0.005). 55% of responders had marked weight gain (>10%) vs. 0% of deteriorators (*p* < 0.001)
			CLZ (*N* = 38)			
			RIS (N = 39)			
			HAL (*N* = 36)			
						- CLZ: Controlling for baseline psychopathology and baseline body weight: partial *r* = 0.39 *p* < 0.02 (total), partial *r* = 0.39; *p* = 0.018 (PANSS general psychopathology), partial *r* = 0.31; *p* = 0.07 (PANSS pos. symptoms), and no significant correlation for PANSS neg. symptoms subscale. WG difference between responders and deteriorators: 5 kg (*p* = 0.002). 40% of responders had marked weight gain (>10%) vs. 0% of deteriorators (*p* = 0.02).
						- RIS: partial *r* = 0.00; *p* = NS. Responders did not gain significantly more than deteriorators.
						- HAL: partial *r* = 0.30; *p* < 0.06. Responders did not gain significantly more than deteriorators.
Ascher-Svanum et al., 2005a	SCZ	248	OLZ (*N* = 187)	6	BPRS	- OLZ: *r* = 0.43; *p* < 0.001; slope = 0.107 kg/unit. Responders had significantly greater WG than deteriorators: 4.71 kg vs. 1.17 kg; *p* < 0.001. Adjusting for treatment duration:partial *r* = 0.21; *p* = 0.003; slope = 0.064 kg/unit, 4.03 kg vs. 2.24 kg; *p* = 0.029.
			PBO (*N* = 61)			
						- PBO: r = 0.36; p = 0.004; slope = 0.052 kg/unit, 0.40 kg vs.−1.83 kg; *p* = 0.003. Adjusting for treatment duration: partial *r* = 0.41; *p* = 0.001; slope = 0.072 kg/unit, 0.80 kg vs.−2.29 kg; p < 0.001.
Ascher-Svanum et al., 2005b^1^	SCZ, SAD, SPF	1996	OLZ (*N* = 1336)	6	PANSS,BPRS, MADRS, SF-36	- OLZ, HAL: Controlling for group, gender, group-gender interaction, baseline weight, baseline psychopathology and treatment duration: BPRS pos. and neg. symptoms: *B* = 0.038; *p* < 0.001. Depressive symptoms (MADRS, BRPS): *B* = 0.030; *p* < 0.001. Mental functioning (SF-36): *B* = 0.026; *p* = 0.047. Physical functioning (SF-36): *B* = 0.028; *p* = 0.021). No gender effect.
			HAL (*N* = 660)			
						- OLZ: Responders vs. deteriorators (BPRS): 2.49 kg vs. 1.42 kg; *p* < 0.001 (BPRS), 2.37 kg vs. 0.59 kg; *p* < 0.001 (PANSS). Controlling for baseline weight, treatment duration and baseline psychopathology: partial *r* = 0.15; p < 0.001 (PANSS). Controlling for baseline weight and baseline psychopathology: partial *r* = 0.24; *p* < 0.001 (PANSS)
						- HAL: Responders vs. Deteriorators: 0.08 kg vs.−0.44 kg; *p* = 0.087 (BPRS), 0.15 kg vs.−0.55 kg; *p* = 0.01 (PANSS). Controlling for baseline weight, treatment duration and baseline psychopathology: partial *r* = 0.11; *p* = 0.006 (PANSS). Controlling for baseline weight and baseline psychopathology: partial *r* = 0.10; *p* = 0.013.
Kinon et al., 2005,^1^	SCZ, SAD, SPF	1191	OLZ	6	BPRS	- CS-WG (*N* = 183) vs. nonCS-WG (*N* = 1008): 17.1 (11.1) vs. 21.0 (12.5); *p* = 0.0009 (BPRS at 6 weeks), 54% vs. 45%, *p* = 0.03 (% of BPRS < 18 at 6 weeks)
Müller et al., 2005^2b^	SCZ, SAD	59	CLZ, HAL, OLZ, RIS	14	PANSS	•[-]ρ = 0.51, *p* < 0.001.
Zipursky et al., 2005	SCZ, SAD, SPF^a^	263	OLZ (*N* = 131)	96	PANSS	- *r* = 0.21; *p* = 0.02. Correlation only seen in week 1 and 6 for both treatment groups (week 12 onward the correlation was NS).
			HAL (*N* = 132)			
**Procyshyn et al.**, **2007**	SCZ	55	RIS, PBO	8	PANSS	- WG not associated with improvement of PANSS total, pos. or neg. subscores (p ≥ 0.1). Controlling for WG: TG (mmol/L): *B* = 3.74, β = 0.285, *p* < 0.037 (total), *B* = 1.565, β = 0.325; *p* = 0.017 (neg. symptoms). TC: *B* = 2.36, β = 0.370; *p* = 0.007 (neg. symptoms). TG, TC did not correlate with pos. symptoms (*p* > 0.25). Responders vs. nonresponders had significantly greater increases in TG (0.8 mmol/L; *p* = 0.004) and TC (0.52 mmol/L; *p* = 0.008) but not weight (*p* = 0.917).
Hermes et al., 2011^b^	SCZ	865 (1245 mixed model)	OLZ,	72	PANSS	- CS-WG vs. nonCS-WG: decrease of 14.1 (15.7) points vs. 5.6 (16.2) points; *p* = NR. Controlling for baseline psychopathology, baseline BMI, treatment group, age, illness duration and investigator site: 12 weeks: partial R2 = 0.01; *p* < 0.001; slope = 0.28 (points/%BMI), 72 weeks: slope = 0.21; *p* < 0.001. No significant differences were found between treatment groups in estimating correlation between PANSS and BMI. No association found between PANSS and total cholesterol or triglycerides.
			RIS,			
			QTP,			
			ZIP,			
			PPZ			
						- OLZ: 12 weeks: partial R2 = 0.02; p = 0.010; slope = 0.37, 72 weeks: slope estimate = 0.17; *p* = 0.001
						- RIS: 12 weeks: NS, 72 weeks: slope = 0.24; *p* = 0.002
						- QTP: 12 weeks: NS, 72 weeks: slope = 0.18; *p* = 0.017
						- ZIP: 12 weeks: NS, 72 weeks: slope = 0.31; *p* = 0.005
						- PPZ: 12 weeks: partial R2 = 0.02; *p* = 0.022; slope = 0.56, 72 weeks: NS
Kemp et al., 2013	SCZ^a^	107	OLZ (*N* = 72)	6	BPRS	- *r* = 0.31; *p* < 0.01 (%BPRS,%WG), *r* = 0.29; *p* = 0.015 (%BPRS/BMI z-score). Controlling for age, gender, race, age at illness onset, BMI z-scoreΔ, baseline BMI z-score, blood pressure, HDL, LDL, BPRS, and CGI: only BMI z-score predicted WG (*p* = 0.01). Became NS when controlling for duration of treatment (*p* = 0.12). TB difference between CS-WG and nonCS-WG: 13.7%; *p* = 0.04. CS-WG did not predict higher rates of response (≥ 30% improvement in BPRS), early response (≥20% at 2 weeks) or remission.
			PBO (*N* = 35)			
						- PBO: *r* = 0.31; *p* = 0.08 (%BPRS/%WG), *r* = 0.33; *p* = 0.06 (%BPRS/BMI z-score). TB difference CS-WG vs. nonCS-WG: 17.3%; *p* = 0.36

a *Population was completely or partially antipsychotic naïve*.

b *Study took measures to ensure compliance*.

1 *All analyzed (Tollefson et al., 1997)*.

2 *Both analyzed (Volavka et al., 2002)*.

*Bolded trials do not show evidence for an AIWG-TB association. The bolded and italicized trial shows evidence for an inverse AIWG-TB association*.

*All correlations where therapeutic benefit (TB) increases with WG/metabolic changes are shown as positive for simplicity. If not specified, correlation is between Δ total scores and absolute WG (kg). Response is ≥ 20% decrease in score unless otherwise specified, nonresponse is anything less than this and deterioration is any increase in psychopathology. CS-WG is ≥ 7% BMI/body weight, unless otherwise specified. Absolute WGs and scores represent averages (1SD). Significance is p ≤ 0.05. APs separated by commas were analyzed together*.

*Pos, positive; neg, negative; NR, Not reported; NS, nonsignificant; WG, weight gain; CS-WG, clinically significantly weight gain; TB, therapeutic benefit; TG, triglyceride; TC, total cholesterol; SCZ, schizophrenia; SAD, schizoaffective disorder; SPF, schizophreniform disorder; BAD, bipolar disorder; AP, antipsychotics; AAP, atypical antipsychotics; TAP, typical antipsychotics; CPZ, chlorpromazine; PBO, placebo; OLZ, olanzapine; CLZ, clozapine; RIS, risperidone; HAL, haloperidol; QTP, quetiapine; ZIP, ziprasidone; PPZ, perphenazine; FPX, flupenthixol; CDX, chlordiazepoxide; TDZ, thioridazine; FLX, fluoxetine; VAR, variety of antipsychotics; SANS, Scale for Assessment of Negative Symptoms; SAPS, Scale for Assessment of Positive Symptoms*.

We identified three double-blind, randomized control trials (DBRCTs) (Planansky, 1958; Holden and Holden, 1970; Poyurovsky et al., 2002), 12 non-randomized prospective trials (Leadbetter et al., 1992; Hummer et al., 1995; Jalenques et al., 1996; Bai et al., 1999; Garyfallos et al., 2003; Lane et al., 2003; Meltzer et al., 2003; Zhang et al., 2003, 2004; Venkatasubramanian et al., 2010; Schwarz et al., 2012; Konarzewska et al., 2013), 13 *post-hoc* analyses of DBRCTs and/or prospective trials (Bustillo et al., 1996; Basson et al., 2001; Czobor et al., 2002; Ascher-Svanum et al., 2005a,b; Müller et al., 2005; Zipursky et al., 2005; Procyshyn et al., 2007; Hermes et al., 2011; Agid et al., 2013b; Kemp et al., 2013), as well as, six chart reviews (Planansky, 1958; Lamberti et al., 1992; Umbricht et al., 1994; Bai, 2006; Hung et al., 2010; Sharma et al., 2011). In two cases, multiple reports analyzed the same original study: three articles (Basson et al., 2001; Ascher-Svanum et al., 2005b; Kinon et al., 2005) analyzed sample A (Tollefson et al., 1997), and two articles (Czobor et al., 2002; Müller et al., 2005) analyzed sample B (Volavka et al., 2002). Therefore, our analysis considered the results of these studies together rather than independently. Finally, one article combined two separate trials which were included individually in our analysis (Basson et al., 2001). Altogether, 31 independent study populations were considered in our analyses below. Table 2 summarizes the proportion of studies which showed evidence for an association.

**Table 2:** Evidence of association for different study variables.

**Variables**	**Evidence for WG, TB link**	
All Studies	22/31	71%
**TREATMENT GROUP**		
OLZ	7/9	78%
CLZ	7/10	70%
RIS	3/6	50%
HAL	1/4	25%
PBO	2/4	50%
**POPULATIONS**		
AP naïve	9/12	75%
East Asian ancestries	4/6	67%
**STUDY TYPE**		
DBRCT	2/3	67%
Prospective study	8/12	67%
*Post-hoc* analysis	11/13	84%
Chart review	4/6	67%
**STUDY DESIGN**		
LOT > 12 weeks	10/15	67%
LOT ≤ 12 weeks	15/21	71%
Compliance ensured	13/18	72%
Control for BBMI	9/10	90%
Control for baseline psychopathology and BBMI	6/7	86%

*AP, antipsychotic; BBMI, baseline body mass index; CLZ, clozapine; DBRCT, doubl-blind, randomized, controlled trial; HAL, haloperidol; LOT, length of treatment; OLZ, olanzapine; PBO, placebo; RIS, risperidone; TB, therapeutic benefit; WG, weight gain*.

### Primary aim: association between weight gain/metabolic changes and therapeutic benefit

#### Weight gain

The 31 independent studies comprised of 6063 individuals being treated with various antipsychotics (CLZ, OLZ, risperidone, haloperidol, quetiapine, ziprasidone, perphenazine, chlorpromazine, flupenthixol, chlordiazepoxide, and thioridazine). Study length ranged from 4 to 384 weeks. Weight change was the most commonly reported metabolic effect; 22 of the 31 studies examining AIWG demonstrated evidence for an association between weight gain and symptom improvement. Eight studies found no significant association and only 1 study showed evidence for the reverse relationship (i.e., weight gain correlating with clinical deterioration). Examining the studies according to study design, DBRCTs and prospective trials demonstrated roughly the same proportion of studies which found an association (67%), while *post-hoc* analyses showed a somewhat higher proportion (80%) (summarized in Table 2).

#### Other metabolic changes

Six studies investigated metabolic parameters other than weight gain, including serum lipids (Garyfallos et al., 2003; Procyshyn et al., 2007; Hermes et al., 2011), leptin (Venkatasubramanian et al., 2010; Schwarz et al., 2012), and insulin (Schwarz et al., 2012; Konarzewska et al., 2013). Garyfallos et al. (2003) found an association between elevated triglycerides (but not total cholesterol) and treatment response in patients receiving OLZ, but not risperidone. Procyshyn et al. (2007) adjusting for weight gain, reported that risperidone treated patients who improved in total and negative symptom PANSS scores had higher triglyceride and total cholesterol levels. Conversely, in a *post-hoc* analysis of the CATIE trial, Hermes et al. (2011) failed to find an association between fasting lipids and treatment response, even when analyses were conducted according to individual antipsychotic.

Other metabolic parameters examined in the context of treatment response included leptin and insulin. Higher serum leptin was an indicator of clinical improvement in two independent studies (Venkatasubramanian et al., 2010; Schwarz et al., 2012), across a variety of different antipsychotic treatments. Schwarz et al. (2012) showed a similar relationship with serum insulin where higher levels predicted better outcomes. Similarly, higher insulin levels were associated with improved psychopathology in an independent study by Konarzewska et al. (2013) in OLZ-, but not risperidone-treated patients.

### Secondary aim: investigation of study variables (clinical and demographic factors)

In order to further explore the association between AIWG and therapeutic benefit, we performed additional analyses to examine if any clinical or demographic factors might be driving the association between AIWG and therapeutic benefit. The results of the investigated factors of interest are summarized below.

#### Type of antipsychotic medication

CLZ (14 studies), OLZ (19 studies), risperidone (14 studies) and haloperidol (8 studies) were the most commonly used antipsychotics in our examined studies (see Table 1). Across studies examining individual antipsychotics, there was a consistent association between AIWG and clinical improvement with OLZ and CLZ treatment. Seven out of 9 studies examining OLZ individually (Czobor et al., 2002; Garyfallos et al., 2003; Ascher-Svanum et al., 2005a,b; Hermes et al., 2011; Kemp et al., 2013), and seven of ten studies examining CLZ individually (Leadbetter et al., 1992; Bustillo et al., 1996; Jalenques et al., 1996; Bai et al., 1999; Czobor et al., 2002; Meltzer et al., 2003; Bai, 2006), found a significant association.

Risperidone and haloperidol are less frequently reported to demonstrate a significant association between AIWG and psychopathology: three out of six studies for risperidone (Lane et al., 2003; Hung et al., 2010; Hermes et al., 2011), and one out of four studies for haloperidol (Ascher-Svanum et al., 2005b). Furthermore, of the seven trials which examined these different antipsychotics comparatively within the same study, four found an association for CLZ or OLZ, while failing to do so for risperidone or haloperidol (Bustillo et al., 1996; Czobor et al., 2002; Garyfallos et al., 2003; Konarzewska et al., 2013). Conversely, three studies found no difference between these antipsychotics when comparatively examined (Hummer et al., 1995; Ascher-Svanum et al., 2005b; Hermes et al., 2011). In summary, CLZ and OLZ are the two most-studied antipsychotics commonly reported to show an association between AIWG and therapeutic benefit.

#### Weight gain and therapeutic benefit in placebo groups

Prior to the introduction and widespread use of antipsychotics, case reports and expert testimonial purported an association between spontaneous recovery and weight gain (Kalinowsky, 1948; Amdisen, 1964). Two studies included in our review found a relationship between weight gain and clinical improvement in control groups treated with placebo (Holden and Holden, 1970; Ascher-Svanum et al., 2005b). However, two other studies failed to find such a relationship in patients receiving placebo (Planansky and Heilizer, 1959; Kemp et al., 2013). Similarly, a study which grouped and analyzed risperidone-treated patients and placebo treated patients failed to find an overall association between weight gain and psychopathology changes (Procyshyn et al., 2007). In conclusion, the small number of studies investigating improvement on placebo and weight gain demonstrated inconsistent results.

#### Individual domains of psychopathology

The complex etiology underlying schizophrenia pathology is highlighted by the existence of individual symptom dimensions (positive, negative, disorganized, cognitive). Several studies examined specific domains of psychopathology in relation to metabolically-related changes during treatment. Improvement in both positive and negative symptoms appeared to be similarly correlated with AIWG (see Table 2). A small number of studies examined cognition, depressive symptoms or social/physical functioning reporting an association between improvement in these domains and AIWG (Planansky and Heilizer, 1959; Holden and Holden, 1970; Lane et al., 2003; Ascher-Svanum et al., 2005b). In terms of other metabolic changes, one study by Procyshyn et al. (2007) suggested that increased serum lipids may correlate with improvement in negative, but not in positive symptoms. Another study by Konarzewska et al. (2013) reported that higher insulin levels correlated with improvement in positive symptoms. Overall, while these preliminary results are interesting, it appears premature to draw conclusions given the limited number of studies which dissected different domains of psychopathology. Given CLZ's superiority in alleviating symptoms in refractory schizophrenia, it would be particularly interesting to investigate the association with AIWG (and other metabolic indices) and different domains of psychopathology in CLZ treated patients (Kane et al., 1988; McEvoy et al., 2006).

#### Baseline psychopathology

A higher baseline psychopathology score may be associated with greater improvement over time based on regression to the mean. This was in fact supported by two studies included in this review (Müller et al., 2005; Hung et al., 2010). Alternatively, higher baseline psychopathology scores could predict treatment resistance. In order to explore if baseline psychopathology could have an effect on the association between AIWG and therapeutic benefit, we examined only those studies which controlled for baseline psychopathology. When examining these studies, six of seven studies found an association between AIWG and therapeutic benefit (Czobor et al., 2002; Meltzer et al., 2003; Ascher-Svanum et al., 2005b; Hung et al., 2010; Hermes et al., 2011; Kemp et al., 2013). Of note, all of these studies also controlled for baseline BMI.

#### Sex

Clinical data has suggested that females may be more susceptible to antipsychotic-associated weight gain as compared to males (Aichhorn et al., 2007; Gebhardt et al., 2009), although others have failed to demonstrate this association (Basson et al., 2001; Ratzoni et al., 2002). Nonetheless, we explored the effects of sex in the context of an association between AIWG and therapeutic benefit. While two studies found a female-exclusive association between AIWG and therapeutic benefit in patients treated with either CLZ (Bai et al., 1999) or risperidone (Hung et al., 2010), three other studies failed to find a sex-specific effect (Meltzer et al., 2003; Ascher-Svanum et al., 2005b; Bai, 2006). Of note, three studies examining male-only populations showed a correlation between AIWG or insulin levels and improved psychopathology (Planansky and Heilizer, 1959; Holden and Holden, 1970; Konarzewska et al., 2013). Overall, sex did not appear to emerge as a critical predictive factor for an association between AIWG and therapeutic benefit.

#### Duration of previous exposure to antipsychotics

First/early exposure to antipsychotics has emerged as an important risk factor for the magnitude of AIWG observed in association with antipsychotics (Correll, 2009). Moreover, response rates are high; ~70% of individuals experiencing remission to the initial antipsychotic-trial (Agid et al., 2013a). In this regard, first episode populations may represent a unique population, as compared to chronic schizophrenia patients, where: (1) response rates to a new antipsychotic trial are expected to be lower; and (2) where a ceiling effect (or plateau) for weight gain may be reached. We identified 12 studies that included antipsychotic-naïve individuals (Planansky, 1958; Planansky and Heilizer, 1959; Holden and Holden, 1970; Poyurovsky et al., 2002; Garyfallos et al., 2003; Lane et al., 2003; Zhang et al., 2003, 2004; Zipursky et al., 2005; Venkatasubramanian et al., 2010; Schwarz et al., 2012; Kemp et al., 2013), of which nine demonstrated a significant association between AIWG and improvement in psychopathology (Planansky, 1958; Planansky and Heilizer, 1959; Holden and Holden, 1970; Garyfallos et al., 2003; Lane et al., 2003; Zipursky et al., 2005; Venkatasubramanian et al., 2010; Schwarz et al., 2012; Kemp et al., 2013) and three did not (Poyurovsky et al., 2002; Zhang et al., 2003, 2004). These results suggest that treatment of early psychosis, a stage of illness where individuals are especially susceptible to weight gain and exhibit high response rates, AIWG, appears to correlate with therapeutic benefit. Notably, duration of prior antipsychotic exposure may also serve as a proxy for younger age, which has been identified as a risk factor for AIWG (Ratzoni et al., 2002). However, only one of the identified studies examined the relationship between treatment response and AIWG specifically in an adolescent population (Kemp et al., 2013). While this study found a significant association, it remains to be determined if developmental trends could independently (i.e., of antipsychotic exposure) mediate an association between treatment response and AIWG.

#### Baseline weight and body mass index (BMI)

Lower baseline weight has been associated with greater AIWG (Basson et al., 2001). Of those studies that controlled for baseline weight or BMI, nine out of ten studies showed an association between weight gain and clinical improvements (Basson et al., 2001; Czobor et al., 2002; Lane et al., 2003; Meltzer et al., 2003; Ascher-Svanum et al., 2005b; Bai, 2006; Hung et al., 2010; Hermes et al., 2011; Kemp et al., 2013), with only one study failing to find an association (Zhang et al., 2003).

#### Ethnic ancestry

The majority of the identified studies included North American and/or European samples but ethnicities in these population were often not reported or taken into account making it difficult to explore the effect of ethnicity. However, six studies were conducted in patients of East Asian ancestry (Bai et al., 1999; Lane et al., 2003, 200; Zhang et al., 2003, 2004; Bai, 2006; Hung et al., 2010). Four of these six studies provided evidence for an association between AIWG and therapeutic benefit (Bai et al., 1999; Lane et al., 2003; Bai, 2006; Hung et al., 2010) (in two studies this link was sex specific; Bai et al., 1999; Bai, 2006). Nonetheless, based on the limited number of studies available, it is difficult to make a conclusion regarding the effects of ethnic ancestry on the association between AIWG and therapeutic benefit.

#### Length of treatment in study

Fifteen studies analyzed the relationship between AIWG and therapeutic benefit at time points beyond 12 weeks. Ten out of these 15 studies found a significant correlation between AIWG and improvement in psychopathology (Planansky, 1958; Leadbetter et al., 1992; Jalenques et al., 1996; Bai et al., 1999; Czobor et al., 2002; Meltzer et al., 2003; Bai, 2006; Hermes et al., 2011; Sharma et al., 2011; Schwarz et al., 2012). Out of the 21 studies that analyzed data at 12 weeks or less, 15 found a significant association (Holden and Holden, 1970; Bustillo et al., 1996; Basson et al., 2001; Garyfallos et al., 2003; Lane et al., 2003; Meltzer et al., 2003; Ascher-Svanum et al., 2005a,b; Zipursky et al., 2005; Hung et al., 2010; Venkatasubramanian et al., 2010; Hermes et al., 2011; Schwarz et al., 2012; Kemp et al., 2013). This would suggest that length of treatment may not be a critical factor mediating the association between AIWG and therapeutic benefit.

Interestingly, a few studies analyzing data at multiple time points found an association earlier in the study, but not at later time points. In a 96-week study, Zipursky et al. (2005) found an association between AIWG and therapeutic benefit in OLZ- and haloperidol- treated patients in the first 6 weeks of treatment, but not after 12 weeks and beyond. Notably, in this study, the two drugs were not examined separately. Similarly, another study looking at CLZ-treated patients found an association between AIWG and therapeutic benefit at 10 weeks of treatment, but not after 48 weeks (Bustillo et al., 1996). Finally, Hermes et al. (2011) also found that the association changed with duration of treatment but this relationship was dependent on the antipsychotic studied. In pherphenazine-treated patients an association between AIWG and therapeutic benefit was found at 12 weeks, but not 72 weeks. Conversely, in risperidone-, quetiapine- and ziprasidone- treated patients no relationship was seen at the 12-week time point but a statistical significant association was then found at 72 weeks. Therefore, looking at these individual studies more closely suggests that there may in fact be a more nuanced relationship, whereby the association between AIWG and therapeutic benefit depends on duration of treatment, but this may depend on the individual medication examined. One could speculate that drugs with a rapid weight gain trajectory demonstrate an early association between therapeutic benefit and AIWG. Conversely, if agents with a slower weight gain trajectory are examined, the association with therapeutic benefit may be more prominent at later time-points in treatment.

#### Compliance

Compliance can be considered an important confounding factor when studying AIWG and therapeutic benefit. For example, patients who are compliant with treatment are expected to get better, and by virtue of taking the antipsychotic drug, also gain weight. Conversely, non-compliance, if not reported, may lead to a false association between lack of weight gain and poor treatment response. Seventeen studies attempted to control for antipsychotic compliance by either including only inpatients throughout the study or by employing different strategies during the study to monitor compliance. Of these studies, 14 found an association between adverse metabolic effects and therapeutic benefit (Planansky, 1958; Planansky and Heilizer, 1959; Holden and Holden, 1970; Leadbetter et al., 1992; Bai et al., 1999; Czobor et al., 2002; Garyfallos et al., 2003; Lane et al., 2003; Meltzer et al., 2003; Hung et al., 2010; Venkatasubramanian et al., 2010; Hermes et al., 2011; Schwarz et al., 2012; Konarzewska et al., 2013), while three did not (Lamberti et al., 1992; Zhang et al., 2003, 2004). Looking specifically at AIWG in relation to therapeutic benefit, 13 of 14 studies found an association, while one did not (Konarzewska et al., 2013). Taken together, compliance was rarely assessed or controlled for in a systematic manner in available studies, despite potential to influence results toward a false negative association. That said, most studies employing strategies to minimize this potential confounder appear to support the existence of a relationship between AIWG and therapeutic benefit.

### Clinical significance

The importance of addressing what constitutes a clinically meaningful relationship between change in weight and psychopathology has been highlighted previously (Hermes et al., 2011). Few of the studies provide sufficient data to assess how much of the variance in psychopathology change can be accounted for by AIWG. The accepted definition of clinically significant weight gain is a 7% or more increase in weight which corresponds to an absolute increase of at least 5 kg or 1.5 kg/m (Allison and Casey, 2001) in a 75 kg person with a BMI of 22. A decrease in psychopathology that is clinically meaningful was often defined in the studies we reviewed as a decrease of 20% or more in the BPRS or PANSS scale, improvement on CGI or a prolonged period of clinical stabilization.

Based on these definitions, five studies suggest a clinically relevant difference in weight gain or change in BMI between those with clinical response and those with poorer response or deterioration. Responders in these studies gained 5.2–9.3 kg more weight and, in one study, BMI increased by 1.7 kg/m^2^ more as compared to non-responders. Response rates were defined as ≥20% decrease in PANSS (Czobor et al., 2002), CGI-I 1-2 (Bai et al., 1999; Bai, 2006), late vs. early relapse (Schwarz et al., 2012) and in one older study, improvement on “gestalt” evaluation (Planansky, 1958).

Three other studies reported weight gain below the defined “clinically meaningful” threshold. One of these studies reported a weight gain difference of 3.5 kg in responders (≥20% decrease in BPRS) as compared to those with clinical deterioration (Ascher-Svanum et al., 2005b). Two other studies demonstrated comparative differences in weight gain of <1.5 kg between responders (defined as ≥20% decrease in PANSS and absolute BPRS score ≤ 17, respectively) and non-responders (Basson et al., 2001; Lane et al., 2003).

Several studies reported the degree of improvement in psychopathology in only those individuals who gained a “clinically meaningful” amount of weight. Kemp et al. (2013) demonstrated a 45.6% decrease in BPRS scores in those who gained ≥7% of their baseline weight, compared with a 31.9% reduction in those without a clinically significant weight gain. Five other studies suggested “subclinical” decreases in PANSS or BPRS (i.e., ≤ 12%) in patients who gained >7% of their body weight (Holden and Holden, 1970; Leadbetter et al., 1992; Ascher-Svanum et al., 2005b; Bai, 2006; Hermes et al., 2011). Taken together, according to the strictest definitions of treatment response and weight increase, only five of 13 studies showed a clinically meaningful association between AIWG and therapeutic benefit, providing mixed evidence that clinically significant weight gain is associated with a clinically meaningful treatment response.

## Discussion

The literature to date suggests that a correlation exists between AIWG and therapeutic benefit, supporting findings from an earlier review (Mackin et al., 2005). In addition, we also identified a modest number of studies suggesting an association between increased serum lipids, leptin, insulin and improvements in psychopathology. We also assessed studies according to variables postulated to influence the AIWG-therapeutic benefit association. Examining studies according to some assurance of medication compliance, according to sex, ethnicity, or prior antipsychotic exposure did not conclusively emerge as moderators between AIWG and therapeutic benefit.

In contrast, four variables emerged as potential modulators of the AIWG-therapeutic benefit association. Whenever BMI and/or baseline psychopathology were controlled for, studies appeared to more consistently find an association. Persistence of the association between AIWG and therapeutic benefit in studies controlling for a “regression to the mean” effect, might argue for a true, rather than spurious association between the two phenomena. Another possible explanation for why studies including these covariates were more likely to demonstrate an association may be that controlling for these variables acts as a proxy for comprehensive study methodology. Furthermore, the combination of duration of treatment with weight gain trajectory associated with specific antipsychotics emerged as a potentially mediating variable. For example, we found some evidence that the highest metabolic liability agents (i.e., OLZ and CLZ) which have rapid weight gain trajectories showed an AIWG-therapeutic benefit association more consistently earlier-on in treatment than lower metabolic liability antipsychotics which have a slower weight gain trajectory. Finally, and notably, the individual antipsychotic examined appeared to influence the AIWG-therapeutic benefit association: those published studies looking at highest metabolic liability antipsychotics, specifically OLZ and CLZ, appeared more likely to show an association between weight gain and improvements in psychopathology, whereas those employing risperidone or haloperidol reported an association less frequently.

### Explaining the link between AIWG and therapeutic benefit

**Figure 2:**
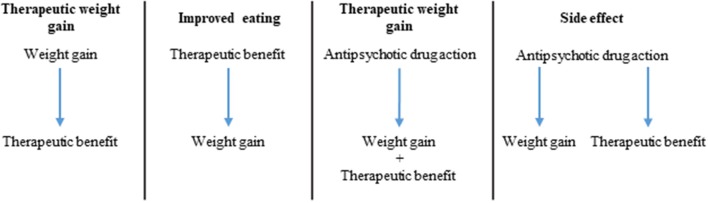
Potential causal pathways for correlation of AIWG and therapeutic effect.

If indeed there is a correlation between AIWG and therapeutic efficacy, an important question becomes: what is the causal nature of this relationship? There are three potentially overlapping explanations for this association: (1) weight gain directly causes clinical improvement; (2) clinical improvement directly causes weight gain; (3) antipsychotic activity of the drugs causes both AIWG and therapeutic benefit by a) common/interdependent or b) independent/mutually exclusive pathways (Figure 2). Possibility 3A would imply that AIWG is necessary for therapeutic efficacy. Possibility 3B would suggest AIWG is a side effect which can be safely targeted (i.e., via lifestyle or pharmacological interventions) without compromising treatment efficacy.

Observations from studies in this review and others suggest that AIWG and therapeutic benefit may occur as a result of the pharmacodynamic properties of antipsychotics but also that their relationship is not necessarily interdependent. For example, all antipsychotics can cause some degree of weight gain with substantial inter-individual variability and therapeutic benefit; however, as outlined in our review, not all of these agents show convincing evidence for an AIWG-therapeutic benefit correlation. This argues against weight gain being necessary for therapeutic benefit. The hypothesis of weight gain as a causal factor for clinical improvement is also weakened by a body of literature linking obesity, metabolic syndrome and insulin resistance to cognitive deficits and poor functioning both in the general population and in schizophrenia (Friedman et al., 2010; Lindenmayer et al., 2012). Finally, a number of lifestyle modification (Brar et al., 2005; Centorrino et al., 2006; Poulin et al., 2007) and drug trials (Maayan et al., 2010) have shown that weight gain prevention or attenuation in patients with schizophrenia treated with SGAs, is not associated with a detrimental effect on psychopathology. Similarly, switching strategies from a high risk to a lower metabolic liability antipsychotic do not suggest substantial risk of clinical deterioration (Mukundan et al., 2010). Taken together, these observations would argue against a direct causal connection between AIWG and therapeutic benefit, or at the very least, suggest that weight gain is not an absolute requirement to observe treatment benefits. Similarly, if recovery in schizophrenia patients is directly related to weight gain, then those who spontaneously recover should gain weight, which was equivocal in the control or placebo treated groups examined here (Holden and Holden, 1970; Ascher-Svanum et al., 2005b; Procyshyn et al., 2007; Kemp et al., 2013).

However, it is not unreasonable to hypothesize that other metabolic pathways both directly, and indirectly associated with weight gained on antipsychotic medications may have specific causal mechanisms with respect to improvements in psychopathology. Hypothetically, some of these pathways may be associated by proxy to weight gain, and explain why AIWG on its own in relation to clinically significant improvements psychopathology is not consistently replicated across studies. For example, antipsychotic-induced glucose dysregulation is well-established in rodent and human models to occur independently to AIWG through “direct” molecular pathways (Houseknecht et al., 2007; Sacher et al., 2008; Smith et al., 2008; Vidarsdottir et al., 2010; Albaugh et al., 2011; Roerig et al., 2011; Hahn et al., 2013; Wu et al., 2014). Moreover, schizophrenia is a heterogeneous disorder, and it may be that unknown, genetic predisposing factors determine which subset of patients may, for example, rely on pathways overlapping with AIWG (or other antipsychotic-induced metabolic effects) for treatment response.

#### AIWG: a proxy for other metabolic pathways mediating antipsychotic-response?

Intriguingly, insulin receptors are expressed in many brain areas and play a role in neuronal growth and synaptic plasticity (Chiu et al., 2008). Abnormalities in brain insulin signaling and reduced brain expression of insulin receptors are reported in schizophrenia (Zhao et al., 2006), and antipsychotics may also impact these pathways (Girgis et al., 2008). Animal work suggests in turn that insulin can activate receptors in the brain inhibiting dopamine synthesis and release in areas implicated in schizophrenia psychopathology, such as the striatum (Liu et al., 2013). Meanwhile, there is evidence from cross-sectional studies to suggest that hyperinsulinemia (associated with peripheral insulin resistance) is linked with reductions in positive and negative symptoms in schizophrenia patients (Fan et al., 2006; Kirkpatrick et al., 2009; Chen et al., 2013; Zhang et al., 2015). In healthy controls treated with antipsychotics, there is evidence to suggest that OLZ increases serum insulin levels and simultaneously induces peripheral insulin resistance, even prior to any weight gain (Hardy et al., 2007; Teff et al., 2013). Interestingly, it has been demonstrated that peripheral insulin resistance precedes central resistance (Brennan, 1987; Banks et al., 2012).

Thus, high insulin levels may be beneficial to the brain early on in the context of hyperinsulinemia and peripheral insulin resistance. Moreover, although weight gain may cause insulin resistance and hyperinsulinemia, hyperinsulinemia can also drive weight gain (Heller, 2004). This could also offer an interesting speculative explanation as to why OLZ and CLZ, two drugs with the most pronounced effects on glucose homeostasis, are most frequently reported to show associations between therapeutic benefit and AIWG, and furthermore demonstrate this association most consistently *early on* in treatment. Although more evidence is required, the two studies included in this review examining insulin levels found that increases in insulin concentrations with antipsychotic treatment were correlated with clinical improvement. Interestingly, one of these investigations found the relationship only in patients treated with OLZ, but not risperidone (Konarzewska et al., 2013). Overall, the current evidence may point to insulin dysregulation as a potential mechanism through which antipsychotics, in particular OLZ and CLZ, are both metabolically and therapeutically active.

#### Clinical implications

As alluded to in earlier sections, confirmation of a true association between AIWG (or other related metabolic risk factors) and therapeutic response in schizophrenia would have important clinical implications. If a degree of metabolic dysregulation is necessary for therapeutic effects in a subset of patients, this may mean that as clinicians we may have to accept that a certain trade-off between cardiovascular health and mental health is inevitable. By the same token, an early and rapid onset of metabolic changes could potentially be used as a marker of good clinical response. Identification of specific metabolic pathways with overlap to treatment response may be exploited to develop more efficacious treatments.

Despite these potential implications, the available literature examining the association between antipsychotic-induced metabolic dysregulation and therapeutic benefit does not allow us to draw firm conclusions as to the clinical significance of what appears to suggest a “statistical,” and somewhat variable association noted mainly between AIWG and treatment response. Thus, given our current state of knowledge, the following observations should be emphasized: (1) AIWG, insulin resistance, and dyslipidemia are considered modifiable, and treatable cardiovascular risk factors; (2) these should be screened for and treated at minimum according to current guidelines (American Diabetes Association, 2004); (3) lifestyle modification and pharmacological intervention trials in this population have demonstrated successful weight loss without clinical deterioration (Tschoner et al., 2007; Bushe et al., 2009; Miller, 2009).

### Study limitations

It is important to acknowledge several limitations which complicate the interpretation of the current literature examining the association between AIWG and therapeutic benefit. Most available studies are retrospective and did not set out a priori to look for an association between metabolic changes and therapeutic benefit. Furthermore, the magnitude of AIWG was not well reported across studies, while it would have been interesting to consider measures such as the 7% increase as cut-off frequently used. Many also did not correct for multiple testing which greatly increases the likelihood of false positives. This may also explain why there was a higher proportion of *post-hoc* analyses that showed an AIWG-therapeutic benefit association vs. other study types (see Table 2). Similarly, the chance for false positives is elevated in studies with small samples, particularly given that power was often not reported. Finally, the studies were quite heterogeneous as a group which impedes straightforward comparisons.

The methodology of this review has limitations as well. Given the exploratory nature of this review, the lack of higher quality prospective RCTs examining clinically meaningful weight gain/psychopathology change, and the heterogeneity of the studies (design, population demographics, antipsychotics used, illness severity), we chose not to undertake a meta-analytical approach. We also did not control for a publication bias in our sample of studies which may have skewed our results in favor of a positive result (i.e., in favor of an association between weight gain and therapeutic benefit).

## Conclusions and future work

In conclusion, this review supports an association between AIWG and therapeutic benefit in patients with schizophrenia, but evidence in terms of clinical significance remains sparse. Additionally, this link was weighted toward patients treated with OLZ and CLZ suggesting these medications possess a shared unique mechanism of action that may relate to their metabolic liability. In this regard, we speculate that the unique effects of CLZ and OLZ on insulin dysregulation could possibly indirectly explain the link between AIWG and therapeutic benefit in a subset of patients. Further research should continue to explore the links between therapeutic benefit and metabolic health with emphasis on both pre-clinical work (to examine mechanisms, with special attention on overlapping metabolic and therapeutic pathways in the CNS), and well-designed prospective clinical trials examining metabolic parameters associated, but also occurring independently to AIWG. Evidence from genetic studies examining genes implicated in energy homoeostasis or appetite/satiety regulation and also overlapping with clinical improvement may also provide clues as to shared pathways. More recent results have also pointed to a role of the gut microbiome in the etiology of schizophrenia and in antipsychotic treatment outcomes, which warrants further investigation (Kanji et al., 2017). Together, this complementary approach has the potential to lead to novel approaches to improve our current understanding and treatment of psychopathology in schizophrenia.

## Author contributions

AR and VM summarized studies and drafted the manuscript. AC and IG contributed to drafting and revising the manuscript and enhanced succinctness and clarity. DM and MH contributed clinical and basic expertise in AIWG and therapeutic benefit, overseeing the synthesis of extracted information and manuscript drafting and editing.

### Conflict of interest statement

The authors declare that the research was conducted in the absence of any commercial or financial relationships that could be construed as a potential conflict of interest.
